# Acute effects of physical and mental fatigue on time perception in basketball players

**DOI:** 10.3389/fpsyg.2026.1759731

**Published:** 2026-02-04

**Authors:** İsmail İlbak, Stefan Stojanović, Serkan Düz, Cihad Onur Kurhan, Matej Suva, Ladislav Cepicka

**Affiliations:** 1 Institute of Health Sciences, İnönü University, Malatya, Türkiye; 2 Faculty of Sport and Physical Education, University of Niš, Niš, Serbia; 3 Department of Coaching Education, Faculty of Sport Sciences, İnönü University, Malatya, Türkiye; 4 Department of Physical Education and Sport, Faculty of Education, University of West Bohemia in Pilsen, Czechia

**Keywords:** acute fatigue, basketball, cognitive load, physical exertion, time perception

## Abstract

**Introduction:**

Basketball is a fast-paced, cognitively demanding sport in which players must make rapid, time-dependent decisions under physical and mental strain. Despite the well-documented effects of fatigue on performance, its influence on athletes’ perception of time remains insufficiently explored. This study aimed to examine the acute effects of physical and mental fatigue on time perception in basketball players.

**Methods:**

This cross-sectional study included 34 healthy, volunteer male basketball players. The experimental procedure consisted of a familiarization session followed by two experimental conditions. Mental fatigue was induced using the color–word Stroop test, whereas physical fatigue was elicited through a standardized plyometric exercise protocol. Time perception was assessed before and after each fatigue condition across four target durations (6, 12, 18, and 24 s). A 72-hour recovery period between sessions was implemented to control for potential carryover effects.

**Results:**

A significant time × fatigue × duration interaction was found (*p* < 0.05), demonstrating that mental and physical fatigue exerted opposite effects on time perception. Mental fatigue resulted in systematic underestimation of time, indicated by negative shifts in time estimation errors across all target durations. In contrast, physical fatigue led to consistent overestimation, reflected by positive shifts in estimation errors. These effects became more pronounced with increasing target duration, with the largest deviations observed at 18 and 24 seconds.

**Conclusion:**

Time perception in basketball is not a fixed mechanism but a dynamic component of performance influenced by transient cognitive and physiological states. Mental fatigue is associated with a compression of perceived time, whereas physical fatigue leads to an expansion of perceived duration. These findings suggest that second-dependent decision-making in basketball is shaped not only by technical and tactical factors but also by fatigue-related states. Integrating fatigue management strategies and time-awareness training into basketball practice and competition may improve temporal accuracy and decision stability under pressure.

## Introduction

Basketball is a dynamic and fast-paced team sport that continuously challenges players’ physical and cognitive abilities (Price, 2014; Stewart, 2023). By its very nature, the game places a strong emphasis on time and seconds. During each offensive possession, teams are required to execute an effective play within a restricted time frame, typically 24 s, placing constant temporal and strategic pressure on the players (Burns and Dunning, 2009; Oliver, 2011). Consequently, athletes’ ability to perform under time constraints and make rapid, strategic decisions becomes a critical determinant of success.

It is widely acknowledged in the scientific literature that elite athletic performance requires exceptional integration of both physical and mental capacities (Kalén et al., 2021; Scharfen and Memmert, 2019). In high-intensity sports, such as basketball, physical and cognitive skills operate in tandem as core determinants of performance (Price, 2014; Stewart, 2023). Within this framework, the emergence of either physical or mental fatigue during competition may undermine performance and increase the likelihood of error.

Previous studies have shown that physical or mental fatigue can significantly impair passing accuracy, ball speed, and shooting technique compared to non-fatigued conditions (Cao et al., 2022; Erculj and Supej, 2009; Habay et al., 2021; Li et al., 2021). Despite such evidence, relatively little is known about how physical and mental fatigue influence an athlete’s perception of time and underlying cognitive processing (Goudini et al., 2024). Given the central role of timing in basketball, understanding how different forms of fatigue alter time perception represents a crucial research question.

Time perception refers to the awareness of the passage of time and is closely intertwined with environmental, psychological, and physiological processes (Allman et al., 2014; Wittmann, 2013; Wittmann and van Wassenhove, 2009). Although its neural mechanisms remain partially understood (Wittmann, 2013), two dominant theoretical frameworks the Scalar Expectancy Theory (also known as the Pacemaker–Accumulator Model) (Gibbon et al., 1984) and the Striatal Beat Frequency Model (Grondin, 2010; Meck, 1983; Meck and Church, 1983) have been widely used to explain the temporal estimation process (Behm and Carter, 2020). These models emphasize the role of event counting and neurotransmitter regulation in cortical activation and coordination. Both theoretical approaches suggest that time perception is highly sensitive to variations in arousal levels (Allman and Meck, 2012; Grondin, 2010). Under specific conditions, subjective time can be distorted or compressed (Eagleman, 2005). Accurate temporal perception, however, enables athletes to make rapid decisions, execute strategies effectively, and maintain control over offensive and defensive sequences.

Recent sport psychology research has further demonstrated that expert athletes possess advantages in temporal processing and anticipation, particularly under severe time constraints. Meta-analytic evidence based on temporal occlusion paradigms indicates that skilled athletes outperform less-skilled counterparts in anticipating actions, highlighting the importance of refined temporal prediction mechanisms in sport performance (Song et al., 2025; Müller et al., 2024). These findings suggest that efficient time perception and prediction constitute a critical cognitive component of high-level athletic performance.

Mental fatigue, typically induced by prolonged cognitive effort, has been shown to impair attentional resources and executive control. According to attention-based models of prospective timing, reduced attentional allocation to temporal information leads to fewer accumulated temporal cues, which may result in an underestimation of perceived duration (Block and Zakay, 1997; Block et al., 2010). Goudini et al. (2024) demonstrated that physical and mental fatigue differentially affect time perception, with physical fatigue producing significant distortions in temporal estimates compared to mental fatigue and control conditions. Additionally, broader reviews of mental fatigue in sport suggest that sustained cognitive effort can impair perceptual–cognitive performance and decision-making in athletes (Pageaux and Lepers, 2018). In contrast, physical fatigue is primarily associated with heightened physiological arousal and altered interoceptive feedback. Arousal-based models of time perception propose that increased physiological activation may accelerate internal timing mechanisms, leading to an overestimation of elapsed time (Allman and Meck, 2012; Grondin, 2010). Empirical evidence indicates that physical exertion can systematically alter duration judgments, supporting the notion that bodily states influence subjective time perception (Behm and Carter, 2020; Tonelli et al., 2022).

To the best of our knowledge, no previous research has directly compared the effects of both physical and mental fatigue on time perception in basketball players. This gap underscores the originality of the present study. Therefore, the aim of this research was to determine the extent to which acute physical and mental fatigue influence time perception in basketball players.

Based on the theoretical frameworks of time perception and previous empirical findings, it was hypothesized that acute mental and physical fatigue would produce distinct effects on perceived duration. Specifically, mental fatigue was expected to lead to an underestimation of time intervals due to reduced attentional engagement with temporal information, whereas physical fatigue was expected to result in an overestimation of time intervals as a consequence of increased physiological arousal.

## Methods

### Participants

The sample size was determined *a priori* using G*Power software (version 3.1.9.3, Germany). Because the primary outcome of interest was the within-subject change in signed time-estimation error from pre to post, an a priori power analysis was conducted using a paired-samples (matched-pairs) design (*t*-tests: means, difference between two dependent means). With a two-tailed significance level set at *α* = 0.05, a desired statistical power of 1 − *β* = 0.80, and an assumed moderate effect size (dz = 0.50), the required total sample size was calculated as *n* = 34. This approach was adopted as a conservative strategy for determining sample size in the absence of reliable a priori estimates for higher-order interaction effects in repeated-measures ANOVA designs. Accordingly, the study was conducted with 34 healthy male basketball players. Participants were between 18 and 25 years of age, had been actively competing as licensed basketball players for at least 3 years, and had regularly participated in team training sessions at least twice per week during the previous year. At the time of data collection, all participants were actively competing in regional amateur basketball leagues. The sample included players from different on-court positions (guards, forwards, and centers), reflecting a typical positional distribution in basketball teams; however, playing position was not included as an analytical factor in the present study. In addition to training frequency, participants reported a typical weekly training volume of approximately 6–8 h, including team practices and conditioning sessions. None of the participants reported any injuries or health problems within the last 6 months, and all provided voluntary informed consent prior to participation. All athletes were free from neurological, cardiovascular, or musculoskeletal disorders. The demographic characteristics of the participants are presented in Table 1.

**Table 1:** Demographic characteristics of the participants (*n* = 34).

Variable	Mean ± SD
Age (years)	21.35 ± 2.17
Height (cm)	185.41 ± 6.70
Body mass (kg)	78.23 ± 8.51
Licensed experience (years)	6.47 ± 2.27
Weekly training frequency (sessions)	3.50 ± 0.81

As shown in Table 1, the participants represented a relatively homogeneous group of young, trained basketball players with similar anthropometric characteristics and competitive experience, providing a controlled sample for examining the effects of acute fatigue on time perception.

### Experimental design

This study employed a cross-sectional experimental design and was conducted with 34 healthy, volunteer male basketball players. All testing procedures were carried out in a controlled laboratory environment that was quiet, evenly illuminated, and maintained at a constant temperature, free from external distractions. The experimental protocol consisted of three consecutive phases. In the first phase, a short familiarization session was conducted to help participants adapt to the testing procedures; data collected during this session were excluded from the analysis. In the second phase, mental fatigue was induced using the color–word Stroop test, and participants’ time perception was assessed both before and immediately after the task. In the third phase, physical fatigue was elicited through a standardized plyometric exercise protocol, with time perception measurements obtained immediately before and after the exercise session. The order of the fatigue conditions was fixed for all participants, with the mental fatigue session always preceding the physical fatigue session; the order was not randomized or counterbalanced. A 72-h rest period was provided between the mental and physical fatigue sessions to minimize potential carryover effects and ensure adequate recovery. This experimental arrangement allowed for a reliable comparison of the effects of both cognitive and physical fatigue on time perception. The study protocol was reviewed and approved by the Inonu University Health Sciences Scientific Research Ethics Committee (Approval No: 2025/8491, Date: 18-11-2025). Written informed consent was obtained from all participants prior to participation, and all procedures were conducted in accordance with the ethical principles outlined in the Declaration of Helsinki.

### Time perception test

Time perception measurements were conducted in a quiet laboratory environment free from external distractions. Participants were seated upright in a standard chair and provided responses using a custom-designed hand dynamometer connected to the BioPac AcqKnowledge data acquisition system. Prior to testing, each participant’s maximum voluntary contraction (MVC) was determined through three maximal grip trials, and the highest recorded value was used as the reference MVC. During the test, participants were instructed to squeeze the dynamometer when they believed the target duration had elapsed. To minimize muscle fatigue, responses were standardized at approximately 20% of the individual MVC. Before the time perception task, participants were given standardized verbal instructions emphasizing that they should rely on their subjective sense of time and that explicit counting strategies (e.g., silently counting seconds) or other deliberate timing strategies should not be used. No concurrent distractor task was employed, and compliance with the instruction not to count was not formally assessed. In the familiarization phase, participants first observed a 24-s interval twice on a digital screen to calibrate their internal timing sense. They then completed four practice trials with feedback, during which they estimated durations of 6, 12, 18, and 24 s once each and received immediate feedback on accuracy. In the main testing phase, participants estimated the same four durations (6, 12, 18, and 24 s) six times each without feedback. The four target durations were presented in a randomized order within each block for each participant. Each trial began with a standardized visual “Start” cue displayed on the screen, after which participants initiated timing internally. The inter-trial interval was fixed at 1,500 ms. No performance feedback was provided during the main testing phase. All responses were recorded by the BioPac system with millisecond precision. For each duration, the mean of six estimations represented the participant’s time perception performance. Previous studies have demonstrated that this protocol yields high internal consistency (intraclass correlation coefficients, ICC = 0.75–0.85), confirming its reliability for assessing time perception under varying cognitive and physical conditions (Gardner et al., 2023; Graham et al., 2023).

### Mental fatigue protocol (Stroop test)

Mental fatigue was induced using the color–word Stroop test, a well-established paradigm for eliciting cognitive load and demands on inhibitory control. The test was administered via a 15.6-inch laptop computer (ASUS, Taipei, Taiwan) with a 60 Hz refresh rate. Color words (red, blue, green, yellow) were presented in 34-point font size in random order, with each stimulus displayed for 1,000 ms, followed by a 636 ms fixation cross, resulting in an average trial duration of approximately 1.64 s. Participants were instructed to focus exclusively on the color of the word while ignoring its semantic meaning and to respond as quickly and accurately as possible. Responses were recorded via a standard keyboard, with color–key associations introduced beforehand to ensure that both hands could be used symmetrically. The test included congruent (e.g., the word “red” printed in red), incongruent (e.g., the word “red” printed in blue), and neutral conditions (color words replaced by non-color words), all presented in randomized order. A total of 1,100 trials were completed, with the entire task lasting approximately 30 min. All instructions were standardized, and responses were automatically recorded by the experimental software. Previous studies have demonstrated that this protocol effectively induces mental fatigue by heavily engaging attentional control, cognitive inhibition, and executive regulation processes (Slimani et al., 2018; Smith et al., 2016; Stoet, 2010). To verify the successful induction of mental fatigue, subjective ratings of mental fatigue were assessed using a Visual Analogue Scale (VAS; 0–10) before and after the Stroop task. In addition, reaction time (RT) and error rate (%) were analyzed as objective performance indicators.

As shown in Table 2, participants reported a marked increase in subjective mental fatigue following the Stroop test, confirming the protocol’s effectiveness in eliciting cognitive exhaustion. Furthermore, significant increases in reaction time and error rate were observed, indicating a slowing of cognitive processing and a reduction in attentional control. Collectively, these findings confirm that the Stroop task successfully induced mental fatigue, as evidenced by both subjective (VAS scores) and objective (RT and error rate) measures.

**Table 2:** Pre- and post-test values for mental fatigue (VAS), reaction time (RT), and error rate (mean ± SD).

Variable	Pre-test	Post-test
Mental fatigue (VAS, 0–10)	2.18 ± 1.21	6.97 ± 1.45
Reaction time (ms)	715.41 ± 102.63	842.70 ± 118.32
Error rate (%)	5.87 ± 3.20	9.60 ± 4.17

### Physical fatigue protocol

Physical fatigue was induced using a plyometric exercise protocol widely employed in previous research (Chang et al., 2020; Pournot et al., 2011). The protocol consisted of four exercises performed in the following fixed order: Split Squat Jump, Lateral Hurdle Jump, Box Jump, and Burpee Jump. Each exercise was performed for three sets of 30 s at maximal effort, interspersed with 60 s of passive rest, resulting in a 1:2 work-to-rest ratio. All exercises were performed on a non-slip laboratory surface. For the Box Jump, 40 cm platforms were used, and for the Lateral Hurdle Jump, 30 cm hurdles were employed. The Burpee Jump was executed using body weight only, without any additional equipment. Prior to the protocol, participants completed a five-minute general warm-up followed by dynamic stretching exercises. During all sessions, participants’ safety was closely monitored by the researchers, and standardized verbal encouragement was provided throughout. Each exercise lasted approximately 3.5 min (90 s of active work + 120 s of rest), with the total duration of the protocol being around 14 min. Previous studies have demonstrated that short-term, high-intensity plyometric protocols of this nature increase oxidative stress markers, induce neuromuscular fatigue, and lead to measurable performance decrements (Chang et al., 2020; Pournot et al., 2011). In the present study, the development of fatigue was monitored using both subjective and performance-based indicators—namely, ratings of perceived exertion (RPE) and reductions in repetition count across sets. RPE values were assessed using the Borg CR10 Scale, which ranges from 0 (“no exertion”) to 10 (“maximal effort”). This scale provides a standardized subjective measure of exercise intensity and perceived fatigue.

As shown in Table 3, RPE scores increased progressively across sets in all exercises, while repetition counts declined in parallel. RPE values were moderate during the first set and reached high levels by the third, indicating the accumulation of perceived exertion. The concurrent reduction in repetitions confirms a progressive decline in performance capacity, demonstrating that the applied plyometric protocol effectively induced acute physical fatigue within a short duration.

**Table 3:** Mean RPE scores and repetition counts for each set across exercises.

Exercise	Set 1 RPE	Set 2 RPE	Set 3 RPE	Set 1 Reps	Set 2 Reps	Set 3 Reps
Split squat jump	6.50	7.50	8.50	22	19	16
Lateral hurdle jump	7.00	8.00	9.00	24	21	18
Box jump	7.50	8.50	9.50	20	17	14
Burpee jump	7.00	8.50	9.50	18	15	12

### Statistical analysis

All statistical analyses were performed using IBM SPSS Statistics for Windows (Version 26.0; IBM Corp., Armonk, NY, United States). Figures were generated using GraphPad Prism (Version 10.3.1; GraphPad Software, San Diego, CA, United States). Prior to the main analyses, data were screened for completeness and outliers. Descriptive statistics are presented as means and standard deviations. Given that all participants completed both mental and physical fatigue conditions, the data were analyzed using a within-subject framework. To examine the effects of fatigue on time perception, a 2 (time: pre, post) × 2 (fatigue type: mental, physical) × 4 (estimated duration: 6, 12, 18, and 24) repeated-measures analysis of variance (RM-ANOVA) was conducted on the signed time-estimation error. The assumptions of sphericity were evaluated using Mauchly’s test. When violations of sphericity were detected for factors involving the duration variable, Greenhouse–Geisser corrections were applied. Significant main effects and interactions were further examined using estimated marginal means, and results are reported with 95% confidence intervals. Effect sizes for omnibus tests are presented as partial eta squared (*ηp*^2^). All statistical tests were two-tailed, and the level of statistical significance was set at *p* < 0.05.

## Results

The results of the present study are presented in the tables and the figure that follow:

Table 4 presents the means and standard deviations of signed time-estimation error for each time × fatigue × duration condition. Under mental fatigue, post-test values consistently shifted toward negative directions across all durations, indicating a systematic tendency toward underestimation of time following mental fatigue. This pattern was particularly pronounced at 12, 18, and 24, where post-test means were substantially lower than pre-test values. In contrast, under physical fatigue, post-test estimates shifted toward positive values across durations, reflecting a tendency toward overestimation of time. This effect increased progressively with duration and was most pronounced at 18 and 24, where large positive post-test means were observed.

**Table 4:** Means and standard deviations of signed time-estimation error across time, fatigue type, and duration.

Duration	Mental pre (M ± SD)	Mental post (M ± SD)	Physical pre (M ± SD)	Physical post (M ± SD)
6 (s)	0.02 ± 0.58	−0.37 ± 0.71	−0.21 ± 0.56	0.47 ± 0.72
12 (s)	−0.02 ± 0.80	−0.83 ± 1.23	−0.08 ± 1.07	0.32 ± 1.08
18 (s)	0.48 ± 1.15	−0.57 ± 2.02	0.66 ± 1.35	1.64 ± 1.93
24 (s)	0.93 ± 2.18	−0.80 ± 2.42	0.29 ± 2.29	2.91 ± 1.87

Values are presented as mean ± standard deviation (SD). Positive values indicate overestimation, whereas negative values indicate underestimation of time.

Table 5 summarizes the results of the repeated-measures ANOVA examining the effects of time, fatigue type, and duration on time perception. The analysis revealed a significant time × fatigue × duration interaction *F*(2.23, 73.69) = 9.89, *p* < 0.001, *ηp*^2^ = 0.23, indicating that pre–post changes in time perception depended jointly on fatigue type and the duration being estimated. This three-way interaction constitutes the primary outcome of the analysis.

**Table 5:** Changes in time perception before and after mental and physical fatigue at different durations.

Source	df₁	df₂	*F*	*p*	Partial *η*^2^
Time	1.33	33.00	0.46	0.502	0.01
Fatigue	1.33	33.00	85.59	<0.001	0.72
Duration	1.67	86.65	9.46	<0.001	0.22
Time × fatigue	1.00	33.00	89.03	<0.001	0.73
Time × duration	2.10	79.88	0.96	0.399	0.02
Fatigue × duration	2.42	69.33	6.31	<0.001	0.16
Time × fatigue × duration	2.23	73.69	9.89	<0.001	0.23

Given the presence of this higher-order interaction, lower-order main effects and two-way interactions are not discussed in detail here. To clarify the direction and pattern of the interaction effects, follow-up simple-simple effects analyses based on estimated marginal means are presented in Table 6.

**Table 6:** Patterns of pre–post changes in time perception under mental and physical fatigue.

Time	Fatigue	Duration	M	SE	95% CI
Pre	Mental	6 (s)	0.02	0.10	[−0.19, 0.22]
		12 (s)	−0.02	0.14	[−0.30, 0.26]
		18 (s)	0.48	0.20	[0.08, 0.88]
		24 (s)	0.93	0.37	[0.17, 1.69]
Pre	Physical	6 (s)	−0.21	0.10	[−0.40, −0.02]
		12 (s)	−0.08	0.18	[−0.45, 0.29]
		18 (s)	0.66	0.23	[0.20, 1.13]
		24 (s)	0.29	0.39	[−0.51, 1.09]
Post	Mental	6 (s)	−0.37	0.12	[−0.62, −0.12]
		12 (s)	−0.83	0.21	[−1.25, −0.40]
		18 (s)	−0.57	0.35	[−1.28, 0.13]
		24 (s)	−0.80	0.41	[−1.65, 0.04]
Post	Physical	6 (s)	0.47	0.12	[0.22, 0.72]
		12 (s)	0.32	0.19	[−0.05, 0.70]
		18 (s)	1.64	0.33	[0.96, 2.31]
		24 (s)	2.91	0.32	[2.25, 3.56]

Values represent estimated marginal means (M), standard errors (SE), and 95% confidence intervals (CI).

Table 6 presents the estimated marginal means for the significant time × fatigue × duration interaction, illustrating how changes in time perception from pre- to post-test differed as a function of fatigue type and duration. The presence of this three-way interaction indicates that pre–post changes in time perception were not uniform, but instead depended jointly on whether fatigue was mental or physical and on the length of the interval being judged. To clarify this interaction, simple-simple effects were examined separately for mental and physical fatigue conditions. Under mental fatigue, post-test estimates generally shifted in a negative direction relative to pre-test values, indicating a tendency toward underestimation of time following cognitive fatigue. This effect was most pronounced at shorter and intermediate durations (6 and 12), where post-test means were consistently lower than pre-test means. Although greater variability was observed at longer durations (18 and 24), the overall pattern under mental fatigue suggests a compression of perceived time after prolonged cognitive effort. In contrast, under physical fatigue, post-test estimates shifted consistently in a positive direction, reflecting a tendency toward overestimation of time following physical exertion. This overestimation was relatively modest at shorter durations (6 and 12) but increased substantially as duration lengthened. Notably, the largest pre–post increases were observed at 18 and 24, indicating that physical fatigue progressively amplifies perceived duration as temporal demands increase. Together, these opposing pre–post patterns under mental versus physical fatigue provide a clear explanation for the significant time × fatigue × duration interaction observed in the omnibus repeated-measures ANOVA. While mental fatigue appears to compress perceived time, particularly at shorter intervals, physical fatigue exerts an expanding effect on perceived duration that becomes increasingly pronounced at longer intervals. These findings demonstrate that fatigue-related distortions in time perception are both qualitatively different and duration-dependent, underscoring the importance of considering fatigue type and temporal scale when examining perceptual–cognitive performance in sport contexts.

**Figure 1:**
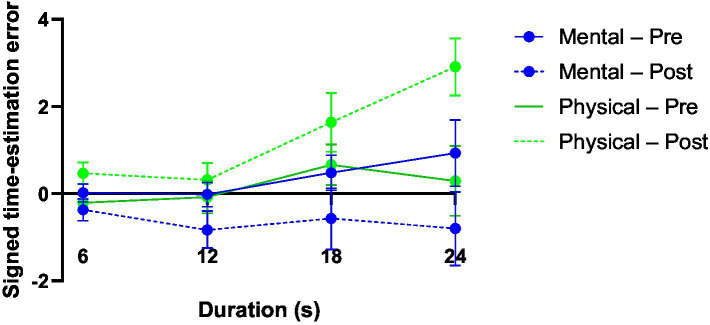
Pre–post changes in signed time-estimation error under mental and physical fatigue across durations.

Figure 1 visually depicts the significant time × fatigue × duration interaction. Under mental fatigue, post-test estimates shift consistently toward negative values relative to pre-test estimates, indicating a tendency toward underestimation of time. In contrast, physical fatigue is associated with a progressive shift toward overestimation at post-test, particularly at longer durations (18 and 24 s). These opposing pre–post trajectories become increasingly pronounced as duration increases, highlighting the duration-dependent nature of fatigue-related distortions in time perception.

## Discussion

The present study aimed to comprehensively examine the differential effects of acute physical and mental fatigue on basketball players’ time perception. The findings revealed that mental and physical fatigue systematically altered time perception in opposite directions. Under conditions of mental fatigue, participants consistently generally underestimated time across all target durations (6, 12, 18, and 24 s). This outcome aligns with both classical and contemporary models, suggesting that cognitive load intensifies the subjective impression that time “passes faster” (Gibbon et al., 1984; Grondin, 2010; Meck, 1983; Meck and Church, 1983). Mental fatigue is known to impair attentional control and increase the demand on cognitive resources directed toward task requirements, consequently distorting temporal evaluation (Csathó et al., 2012; Faber et al., 2012). Although underestimation was evident across durations, this effect was most pronounced at shorter and intermediate intervals, whereas greater variability emerged at longer durations (18 and 24 s). These patterns suggest that participants’ attentional resources were increasingly allocated to high-level cognitive control processes, supporting neuropsychological evidence linking cognitive load to the subjective acceleration of time (Wittmann, 2013; Wittmann et al., 2019).

Moreover, the consistent reports of time passing faster under mental fatigue highlight the central role of attentional distraction and cognitive overload in temporal processing. Sustained cognitive effort likely narrows attentional focus to task-related demands, reducing awareness of the passage of time and leading to compressed temporal judgments. This interpretation is supported by neurophysiological evidence showing reduced attention-related ERP amplitudes under fatigue (Möckel et al., 2015). Collectively, these findings suggest that mental fatigue induces a subjective “acceleration” of time through diminished attentional control and cognitive resource depletion.

In contrast, under physical fatigue, participants showed a clear tendency toward overestimation of time, particularly at longer durations, indicating that increased physiological arousal and autonomic nervous system activation may produce a subjective “slowing down” of time (Hancock, 1993; Pollatos et al., 2014; Schwarz et al., 2013). The overestimation observed at longer durations (18 and 24 s) suggests that physiological arousal–related processes reinforce this perceptual bias. Heightened physiological arousal likely shifts attention from temporal monitoring toward bodily sensations of effort, thereby extending subjective duration (Féry et al., 1997; Goudini et al., 2024). This is consistent with the internal clock model (Treisman, 1963), which posits that increased arousal accelerates the internal pacemaker, leading to perceived time dilation.

Although physiological variables such as heart rate, body temperature, and blood lactate concentration were not directly measured in the present study, prior research suggests that such markers are closely associated with temporal estimation biases during physical exertion (Maci et al., 2025). Furthermore, elevated perceived exertion and attentional shift during high-intensity exercise strengthen the experience of time passing more slowly (Clemente-Suárez et al., 2024). Therefore, time overestimation following physical fatigue likely arises from an integrated mechanism involving both physiological arousal and cognitive resource redistribution.

While modest baseline (pre-test) differences were observed at some durations, the critical finding of the present study lies in the opposing pre–post shifts following mental versus physical fatigue. The systematic pattern of underestimation under mental fatigue and overestimation under physical fatigue suggests complementary yet opposing mechanisms that influence temporal processing. These findings support neurocognitive theories positing that mental fatigue “accelerates” while physical exertion “decelerates” subjective time (Goudini et al., 2024; Marcora et al., 2009; Van Cutsem et al., 2017).

Notably, the absence of a significant main effect of time indicates that pre–post changes in time perception were not uniform across conditions. Instead, the significant time × fatigue and time × fatigue × duration interactions demonstrate that temporal distortions depended strongly on both the type of fatigue and the duration being estimated. The magnitude of the mean pre–post shift increased with longer target durations, suggesting that extended intervals place greater demands on attentional and physiological regulation, thereby amplifying temporal bias (Block and Zakay, 1997; Zakay and Block, 2004). Short intervals are typically governed by automatic processes, whereas longer intervals engage executive and attentional control (Grondin, 2010). Hence, the present findings underscore the multicomponent nature of time perception and the pivotal role of the attention–arousal interaction in temporal estimation.

From a theoretical perspective, the present study extends the literature on time perception by demonstrating that mental and physical fatigue exert not only distinct but also opposing effects on temporal estimation within the same athletic population. While previous studies have typically examined the influence of either cognitive load or physical exertion in isolation, the direct within-subject comparison employed here provides novel evidence that fatigue-related distortions in time perception are fatigue-type specific and duration dependent. This finding advances current models of temporal processing by highlighting the dynamic interaction between attentional control and physiological arousal under ecologically relevant fatigue conditions.

An additional contribution of the present study lies in its focus on trained basketball players, a population for whom temporal precision is a critical performance component. Compared with ordinary adults, basketball players are repeatedly exposed to strict temporal constraints (e.g., shot clock pressure and rapid offensive–defensive transitions), which may foster more refined temporal representations and anticipatory skills. Despite this expertise, the present findings demonstrate that acute fatigue can still systematically distort time perception in athletes, suggesting that even well-adapted temporal systems remain vulnerable to transient cognitive and physiological states. This observation underscores that fatigue-related temporal distortions are not limited to non-athletic populations but also have direct relevance for sport performance.

A Several limitations of the present study should be acknowledged. First, the order of the experimental conditions was fixed, with the mental fatigue session always preceding the physical fatigue session. Although a familiarization phase was included and a 72-h recovery interval was provided between sessions, the absence of randomization or counterbalancing means that order-related effects such as learning, task familiarity, expectancy, or habituation cannot be fully excluded. Consequently, differences observed between mental and physical fatigue conditions should be interpreted with caution. Second, while participants were explicitly instructed not to use counting or deliberate timing strategies during the time production task, no distractor task (e.g., articulatory suppression) or formal compliance assessment was implemented. As a result, the possible influence of covert counting or individual timing strategies cannot be ruled out, limiting strong mechanistic conclusions regarding automatic or purely perceptual timing processes. Third, although both the mental (Stroop task) and physical (plyometric exercise) protocols successfully induced fatigue, the resulting fatigue states were not quantitatively matched in terms of intensity, duration, or underlying physiological and cognitive demands. This constrains direct comparisons regarding the relative magnitude of mental versus physical fatigue effects on time perception. Fourth, the study sample consisted exclusively of male basketball players, which limits the generalizability of the findings to female athletes, other sports, and non-athletic populations. Fifth, time perception was assessed using a laboratory-based time production task under highly controlled conditions. While this approach enhances internal validity and measurement precision, it may not fully capture the dynamic and context-dependent nature of time perception in real-world or competitive sport settings, thereby limiting ecological validity. Finally, although the statistical analyses were corrected to address initial specification issues, remaining analytic constraints such as the modest sample size and reliance on repeated measures within a single cohort may limit statistical power and the generalizability of the inferential conclusions. Future research should address these limitations by employing counterbalanced designs, incorporating objective controls for timing strategies, matching fatigue states across conditions, recruiting more diverse samples, and adopting more ecologically valid assessment paradigms.

## Conclusion

This study demonstrated that acute mental and physical fatigue exert opposing yet systematic effects on time perception. Mental fatigue was associated with a general tendency toward underestimation of time, reflecting a subjective acceleration of temporal experience likely related to attentional depletion and reduced cognitive control, whereas physical fatigue was associated with a shift toward overestimation of time, consistent with a subjective deceleration linked to heightened physiological arousal and interoceptive awareness. Importantly, these effects were duration dependent and varied as a function of fatigue type, highlighting that time perception is not a unidimensional construct but rather a multifaceted phenomenon shaped by the dynamic interplay of cognitive and physiological processes. The interaction between attentional allocation and arousal level emerges as a key determinant of temporal accuracy under fatigue.

From a practical standpoint, the differential effects of mental and physical fatigue have important implications for training design, performance management, and decision-making in sports contexts, particularly in time-constrained environments such as basketball. The present findings suggest that fatigue-related distortions in time perception may influence second-dependent decisions even in trained athletes, underscoring the importance of considering both cognitive and physical fatigue when planning training loads and competitive strategies. Future research should aim to further clarify these mechanisms using designs that incorporate enhanced control of fatigue states and greater ecological validity, thereby contributing to a more comprehensive understanding of fatigue-related changes in perceptual–cognitive performance in sport.

## Data Availability

The data analyzed in this study is subject to the following licenses/restrictions: The dataset is available from the corresponding author upon reasonable request due to ethical restrictions. Requests to access these datasets should be directed to Ladislav Cepicka, lcepicka@ktv.zcu.cz.
